# Spatial Disparities in Mifepristone Use for Early Miscarriage and Induced Abortion Among Obstetrician–Gynecologists Practicing in Massachusetts

**DOI:** 10.1089/whr.2024.0085

**Published:** 2024-10-04

**Authors:** Emily Newton-Hoe, Alisa B. Goldberg, Jennifer Fortin, Elizabeth Janiak, Sara Neill

**Affiliations:** ^1^ Harvard T.H. Chan School of Public Health, Boston, Massachusetts, USA.; ^2^ Department of Obstetrics, Gynecology, and Reproductive Biology, Brigham & Women’s Hospital, Boston, Massachusetts, USA.; ^3^ Harvard Medical School, Boston, Massachusetts, USA.; ^4^ Planned Parenthood League of Massachusetts, Boston, Massachusetts, USA.; ^5^ Department of Obstetrics and Gynecology, Beth Israel Deaconess Medical Center, Boston, Massachusetts, USA.

**Keywords:** mifepristone, geographic disparities, abortion, miscarriage

## Abstract

**Background:**

About 25% of pregnancies end in early miscarriage or abortion annually in the United States. While mifepristone is part of the most effective medication regimen for miscarriage and abortion, regulatory burdens and legal restrictions limit its provision in obstetric–gynecological practice. The extent of geographic disparities in mifepristone use is unknown.

**Objectives:**

We sought to ascertain whether regional “deserts” for mifepristone-based miscarriage and abortion care exist in Massachusetts using geographic regions specified by the Commonwealth’s Executive Office of Health and Human Services.

**Methods:**

We fielded a cross-sectional survey of obstetrician–gynecologists practicing in Massachusetts. We weighted survey data to account for differential nonresponse by provider sex, region, and years in independent practice.

**Results:**

Among obstetrician–gynecologists in independent practice with region data (*n* = 148), 51.0% reported using mifepristone for miscarriage and 43.5% for abortion. Significant differences in reported use were observed across regions (*p* < 0.001 for both indications). Barriers to using mifepristone for miscarriage management also varied across regions. Respondents outside of Boston and Western Massachusetts were more likely to report gaps in knowledge about regulations and prescribing and had less prior experience using mifepristone. In a multivariable model adjusting for provider sex and practice type, obstetrician–gynecologists outside of Boston had significantly lower odds of using mifepristone for miscarriage (adjusted odds ratio [aOR] = 0.14, 95% confidence interval [95% CI] = 0.08–0.25) and abortion (aOR = 0.46, 95% CI = 0.26–0.82), compared to Boston-based obstetrician–gynecologists.

**Conclusion:**

Mifepristone provision varies significantly by Massachusetts region. This may lead to spatial disparities in reproductive health outcomes.

## Introduction

Pregnancy loss (also known as miscarriage) and induced abortion are common in the first trimester, with about 10% of known pregnancies ending in miscarriage and 15% ending in abortion.^1–3^ Therefore, an estimated 25% of pregnancies end in either miscarriage or abortion in the first trimester, a period in which mifepristone can be used for management. The United States Food and Drug Administration (FDA) first approved mifepristone in 2000 for medication abortion.^4^ Together with misoprostol, it is part of the most efficacious evidence-based medication regimens for miscarriage and abortion care.^5–8^ Over 5 million patients in the United States have used mifepristone since 2000, with medication abortion comprising more than half of all abortions.^9^

Although mifepristone is approved by the FDA for abortion care and supported by strong evidence for miscarriage management, regulatory burdens and evolving legal restrictions limit its use across the United States.^10^ For example, mifepristone has been under a restricted distribution program since it received its initial approval in 2000 and regulated by the Mifepristone Risk Evaluation and Mitigation Strategy (REMS) program since 2011.^11^ Under the REMS program, providers must be certified to dispense mifepristone, providers must obtain a signed Patient Agreement Form before use, and the drug must be administered in**-**person at clinics and hospitals.^12^ During the COVID-19 pandemic, the requirement for in-person dispensing of mifepristone was relaxed and then permanently removed.^13^ In 2023, the REMS also changed to allow certified pharmacies to dispense mifepristone.^13^ These changes allowed clinicians to prescribe mifepristone *via* telehealth and to dispense the medication through certified mail-order pharmacies.^14^ Removing these restrictions expands access to people facing structural barriers to care, including those living far from healthcare clinics. However, the limited number of certified pharmacies and ongoing REMS restrictions like prescriber certification and completion of Prescriber and Patient Agreement Forms^13^ prevent mifepristone from being fully accessible for miscarriage management and abortion care.

The lack of FDA labeling of mifepristone for early pregnancy loss (also known as first-trimester miscarriage) limits access to this evidence-based treatment as well. Because using mifepristone for early pregnancy loss is considered an “off-label” use of the drug, clinicians may be hesitant to incorporate mifepristone into their practice. Their concerns may include FDA compliance, uncertainties about how REMS requirements intersect with federal and state abortion laws, and fears of being stigmatized for providing abortion care.^15–17^

State and federal restrictions further limit access to mifepristone. For example, during the COVID-19 pandemic, clinics located in abortion hostile states were less likely to offer innovative service delivery options like dispensing mifepristone curbside, providing mail delivery of mifepristone, or offering telehealth follow-up.^18^ While the US Supreme Court preserved access to mifepristone at the federal level in its decision in *Alliance for Hippocratic Medicine v. FDA* in June 2024, continued legal challenges may exacerbate limited mifepristone access across the United States.^19,20^

Other studies have sought to understand barriers to mifepristone provision at a regional or national scale.^16,17,21–23^ For example, a qualitative study among abortion-trained primary care providers in New England found that REMS requirements and a lack of organizational support were the primary barriers to abortion provision.^21^ An online qualitative survey among primary care providers across the United States reported similar barriers to mifepristone provision, with providers believing that lack of mifepristone in primary care contributed to adverse patient outcomes.^16^ While understanding these barriers at a broad scale is important, so too is recognizing within-state variations in mifepristone use. Given the lack of data on spatial disparities in mifepristone use, we examined geographic variations in mifepristone use in Massachusetts, a state with minimal abortion restrictions and no state-level regulations of mifepristone.^24^ Here, we seek to understand spatial disparities in mifepristone use and barriers to use. We also seek to understand whether geographic “deserts” for mifepristone-based miscarriage and abortion care exist in Massachusetts.

## Materials and Methods

This study is part of a larger mixed-methods study examining mifepristone provision for miscarriage and abortion care among obstetrician–gynecologists in Massachusetts.^23^ We limited the present study to Massachusetts obstetrician–gynecologists who had treated at least one patient for early pregnancy loss in the year prior to the survey period (January to July 2021). We identified obstetrician–gynecologists through the American Medical Association Physician Masterfile, a database of practicing physicians that is routinely used for academic research.

Obstetrician–gynecologists with subspecialties whose scope of practice generally does not include pregnancy loss care, including urogynecology and gynecological oncology, were excluded from the survey. We identified these obstetrician–gynecologists by compiling a list of urogynecology and gynecological oncology specialists in Massachusetts, cross-referencing it with the Masterfile, and removing these specialists from survey distribution. Additionally, at the start of the survey, a screening question asked the respondent to choose their subspecialty. If the respondent chose urogynecology or gynecological oncology, the survey ended, and they were informed they did not meet the study inclusion criteria. Thus, from the 1086 records in the Masterfile as of October 2020, we removed 27 obstetrician–gynecologists who were in practice types that do not generally manage early pregnancy loss or provide abortion care (*i.e.,* urogynecology and gynecological oncology). We also removed 375 obstetrician–gynecologists who had missing or invalid email addresses, for a total fielding sample of 684.

The survey was programmed in REDCap with an estimated burden of 10 minutes. We disseminated the survey *via* email from January 2021 to July 2021 to the 684 eligible obstetrician–gynecologists in the Masterfile. We sent an initial email and up to seven email reminders, for a total of eight email contacts. Obstetrician–gynecologists who completed the survey were offered a $10 Amazon gift card and free access to an online “Medical management of early pregnancy loss” learning module from the Massachusetts Medical Society.

This study was approved by the institutional review board at the Mass General Brigham.

### Measures

Formative qualitative research with Massachusetts-based obstetrician–gynecologists providing early pregnancy loss care helped inform the survey domains.^17^ The survey queried obstetrician–gynecologists on knowledge of mifepristone for medical management of early pregnancy loss, knowledge of the REMS program, use of mifepristone for various clinical scenarios, sufficiency of training related to the management of early pregnancy loss, barriers and facilitators of mifepristone use, and the impact of the COVID-19 pandemic on miscarriage management and abortion care. The survey also included questions on practice setting, education, and sociodemographic characteristics.

The primary outcomes of interest were use of mifepristone for miscarriage management (respondent reported offering “medical management—mifepristone and misoprostol” to treat patients with first-trimester pregnancy loss or miscarriage) and use of mifepristone for abortion care (respondent reported offering “medical management—mifepristone and misoprostol” to treat patients seeking first-trimester abortion).

**FIG. 1.**
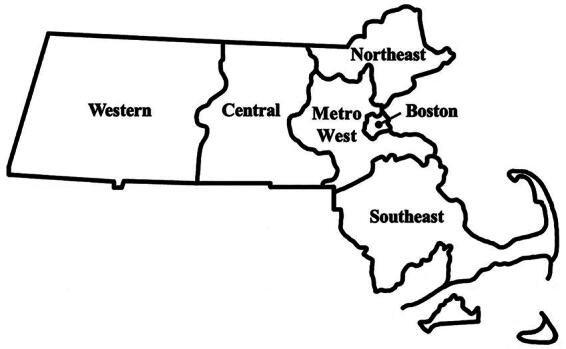
Six geographic regions in Massachusetts, according to the Massachusetts Executive Office of Health and Human Services.^26^

The primary predictor was Massachusetts region. We used the geographic regions as specified by the Massachusetts Executive Office of Health and Human Services (EOHHS),^25^ mapping the zip code associated with the respondent’s primary practice to the six EOHHS regions—Western, Central, MetroWest, Northeast, Boston, and Southeast (Fig. 1). The Boston region is the most populous, with a unique medical ecosystem home to 25 hospitals and 20 community health centers.^26^ The MetroWest region borders Boston and contains several mid-sized cities. The Central and Northeast regions are more suburban with a few mid-sized cities. The Southeast region comprises suburban towns as well as a fair amount of rural regions. The Western region is more rural than the rest of the state. Descriptive analyses assess each region separately, while regression analyses collapse the six regions into Boston (reference group), MetroWest, and other regions (Western, Northeast, Central, and Southeast) based on sample sizes.

We used physician sex and practice type as covariates for this analysis.^27^ Physicians reported their sex according to their sex assigned at birth, as stated on their original birth certificate (response options: male, female). We determined practice type based on the respondent’s current primary practice setting (response options: academic/university faculty, private practice—solo, private practice—group, health maintenance organization (HMO)/staff model/nonuniversity hospital, federally qualified health center (FQHC), community health center or freestanding clinic, or other). Regression analyses collapse the six practice types into academic (reference group), private practice (solo and group), and other (HMO/staff model/non-university hospital, FQHC, community health center or freestanding clinic, and other) based on sample sizes.

Barriers to using mifepristone for miscarriage management were assessed through the survey question: “What factors have made it difficult for you to use mifepristone for early pregnancy loss in your practice?” Respondents were provided with a list of 13 items and could select all that apply. We only asked this survey question to respondents who do not currently offer mifepristone to patients with first-trimester pregnancy loss or miscarriage because we sought to understand the specific challenges or reasons for not using mifepristone in this clinical scenario. We did not ask a question about barriers to using mifepristone for abortion care because the rate of abortion provision was a secondary outcome in this study.

### Data analysis

This analysis is limited to 148 (out of 198) obstetrician–gynecologists who provided region data and are in independent practice (that is, not in residency or fellowship). Descriptive statistics and chi-square tests assess whether the prevalence of mifepristone use for miscarriage management and abortion care varies across regions. Descriptive statistics also investigate whether barriers to mifepristone provision for miscarriage management vary across regions. Finally, a multivariable logistic regression model describes the odds and 95% confidence intervals (95% CI) associated with mifepristone use for miscarriage management and abortion care by region, controlling for primary practice setting and provider sex.^27^ We set statistical significance at alpha ≤0.05.

We used R version 4.2.2. for data cleaning and STATA version 17 for analysis. We applied the raking method^28^ to weight the data for differential nonresponse by physician sex, region, and years in independent practice. We also applied a finite population correction because our sampling frame was a census of obstetrician–gynecologists in Massachusetts.^29^

## Results

A total of 198 obstetrician–gynecologists responded to the survey (response rate = 29.0%). Table 1 presents the demographic characteristics of the 148 obstetrician–gynecologists in independent practice with region data, who are the focus of this analysis. Respondents mostly self-identified as non-Hispanic White (76.4%) and female (64.0%). Most had been in practice for >15 years (59.3%). A small subset of respondents completed fellowship or subspecialty training (16.9%), with the primary fellowship type being family planning (32.9%). Respondents primarily practiced in private (46.3%) or academic (32.2%) settings. The most common caseload of publicly insured patients was <20% (41.7%).

**Table 1.** Demographic Characteristics Among Obstetrician–Gynecologists in Independent Practice in Massachusetts in 2021 (*n* = 148)

Variable	Unweighted *n* (weighted %)
Race/ethnicity	
Hispanic	7 (4.2)
Non-Hispanic White	107 (76.4)
Non-Hispanic Asian	19 (11.3)
Non-Hispanic Black	6 (1.7)
Non-Hispanic two or more races	3 (2.1)
Non-Hispanic other	2 (2.9)
Missing	4 (1.5)
Provider sex	
Male	27 (33.4)
Female	115 (64.0)
Missing	6 (2.6)
Years in practice	
<3 years	20 (10.4)
3–8 years	22 (10.7)
9–15 years	30 (19.6)
>15 years	76 (59.3)
Fellowship or subspecialty training	
Yes	37 (16.9)
No	110 (82.4)
Missing	1 (0.6)
Fellowship type	
Family planning	14 (32.9)
Maternal fetal medicine	12 (28.1)
Reproductive endocrinology and infertility	6 (25.5)
Other	3 (9.7)
Pediatric and adolescent gynecology	2 (3.9)
Practice setting	
Academic/university faculty	70 (32.2)
Private practice—solo or group	47 (46.3)
HMO/staff model, non-university hospital	19 (15.5)
Community health center/FQHC/other	12 (6.0)
Publicly insured patients	
None	3 (3.0)
<20%	58 (41.7)
21%–50%	46 (30.5)
>50%	20 (12.1)
Unsure	21 (12.6)

Fellowship-type question excludes 121 respondents who did not complete fellowship or subspecialty training or had missing data. Percentages may not add up to exactly 100% due to rounding.

FQHC, federally qualified health center; HMO, health maintenance organization.

### Prevalence of mifepristone use for miscarriage management and abortion care

**FIG. 2.**
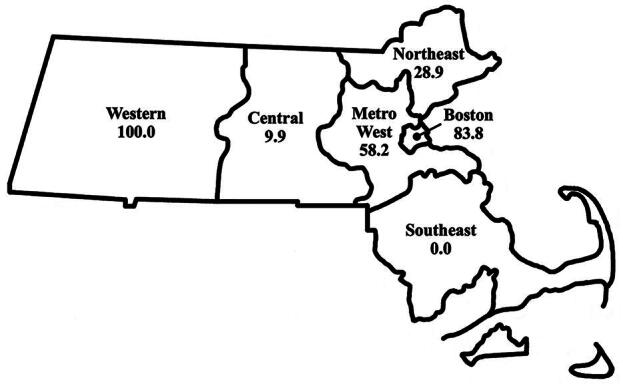
Weighted percent of obstetrician–gynecologists in Massachusetts who used mifepristone for miscarriage management in 2021, by geographic region.

**FIG. 3.**
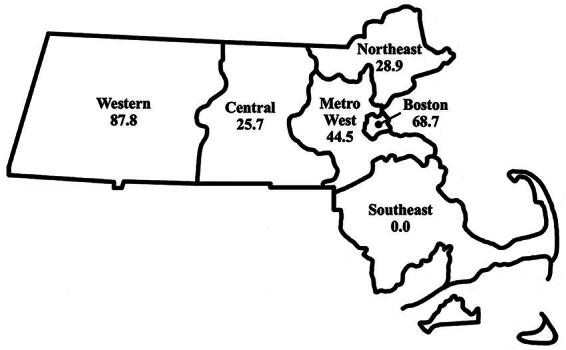
Weighted percent of obstetrician–gynecologists in Massachusetts who use mifepristone for abortion care in 2021, by geographic region.

Just over half (51.0%) of obstetrician–gynecologists reported using mifepristone for miscarriage management and slightly less (43.5%) for abortion care (Table 2). Reported use of mifepristone for both indications was significantly different by region (*p* < 0.001 for both). Respondents in Western, MetroWest, and Boston reported above average mifepristone use for both indications, while respondents in Southeast, Northeast, and Central regions reported below average use (Figs. 2 and 3). The highest reported use for both indications was in the Western region (100.0% and 87.8% for miscarriage management and abortion care, respectively), and the lowest reported use was in the Southeast region (0% for both indications).

**Table 2.** Use of Mifepristone for Miscarriage and Abortion Care by Obstetrician–Gynecologists in Independent Practice in Massachusetts in 2021, by Region (*n* = 148)

Region	Weighted % uses mifepristone for miscarriage management	Weighted % uses mifepristone for abortion care
Overall (*n* = 148)	51.0	43.5
Test of distribution across regions (*p* value)^a^	<0.001	<0.001
Boston (*n* = 66)	83.8	68.7
MetroWest (*n* = 42)	58.2	44.5
Central (*n* = 14)	9.9	25.7
Northeast (*n* = 12)	28.9	28.9
Western (*n* = 7)	100.0	87.8
Southeast (*n* = 7)	0.0	0.0

^a^ Chi-square test was used to test whether the proportion using mifepristone in at least one region differed from at least one other region.

Data weighted to account for differential nonresponse by provider sex, region, and years in independent practice.

A preliminary sensitivity analysis examined practice characteristics in the Western and Southeast regions, given that respondents in these regions answered uniformly and the sample sizes were small (*n* = 7 in each; Table 3). Respondents in the Southeast region were more commonly male (81.5% vs. 33.4%), in private practice (81.5% vs. 46.3%), and had over 15 years of experience (79.8% vs. 59.3%) compared to respondents overall. Respondents in the Western region were more commonly in academic practice (75.1% vs. 32.2%) compared to respondents overall and otherwise similar in terms of provider sex and years in practice.

**Table 3.** Preliminary Sensitivity Analysis of Regional Differences in Practice Characteristics Among Obstetrician–Gynecologists in Independent Practice in Massachusetts in 2021 *(n* = 148)

Variable	Unweighted *N* (weighted %)						
	Overall (*n* = 148)	Boston (*n* = 66)	MetroWest (*n* = 42)	Central (*n* = 14)	Northeast (*n* = 12)	Western (*n* = 7)	Southeast (*n* = 7)
Provider sex							
Male	27 (33.4)	6 (14.4)	8 (28.1)	4 (43.5)	3 (34.9)	1 (20.2)	5 (81.5)
Female	115 (64.0)	57 (83.7)	34 (71.9)	9 (51.7)	9 (65.1)	4 (55.3)	2 (18.5)
Missing	6 (2.6)	3 (1.9)	0 (0.0)	1 (4.8)	0 (0.0)	2 (24.6)	0 (0.0)
Practice type							
Academic	70 (32.2)	54 (79.8)	7 (16.6)	2 (9.9)	1 (9.3)	5 (75.1)	1 (10.8)
Private	47 (46.3)	5 (9.3)	22 (58.3)	7 (56.0)	7 (54.0)	1 (12.3)	5 (81.5)
FQHC	12 (6.0)	6 (9.5)	4 (6.7)	1 (4.8)	1 (8.0)	0 (0.0)	0 (0.0)
HMO	19 (15.5)	1 (1.5)	9 (18.5)	4 (29.3)	3 (28.7)	1 (12.6)	1 (7.8)
Years in practice							
<3 years	20 (10.4)	15 (27.9)	2 (4.5)	1 (5.0)	1 (9.3)	1 (12.6)	0 (0.0)
3–8 years	22 (10.7)	9 (9.7)	7 (12.2)	2 (9.8)	2 (11.6)	2 (25.2)	0 (0.0)
9–15 years	30 (19.6)	12 (15.9)	10 (23.7)	2 (9.8)	3 (21.7)	1 (20.2)	2 (20.2)
>15 years	76 (59.3)	30 (46.6)	23 (59.7)	9 (75.4)	6 (57.4)	3 (42.1)	5 (79.8)

FQHC, Federally Qualified Health Center; HMO, Health Maintenance Organization.

### Barriers to mifepristone use for miscarriage management

The most significant barrier to using mifepristone for miscarriage management was uncertainty about how to comply with the FDA REMS regulations (23.3% overall, ranging from 4.0% in MetroWest to 64.2% in Southeast; Table 4). Other top barriers related to lack of experience with mifepristone, such as not having used it before for any indication (14.4% overall, ranging from 0% in Boston to 27.2% in Central), and being unfamiliar with its clinical data for early pregnancy loss (12.7% overall, ranging from 0% in Northeast and Southeast to 35.0% in Central). Two barriers were not reported by any respondent: (1) uncertainty with managing complications associated with the medical management of early pregnancy loss and (2) concerns about having the patient sign a required consent form from the mifepristone manufacturer.

**Table 4.** Most Significant Barriers to Using Mifepristone for Miscarriage Management Among Obstetrician–Gynecologists in Independent Practice in Massachusetts in 2021, by Region (*n* = 53)

Barrier	Overall (*n* = 53)	Boston (*n* = 9)	MetroWest (*n* = 17)	Central (*n* = 12)	Northeast (*n* = 8)	Southeast (*n* = 7)
Unsure how to comply with FDA REMS regulations	23.3	9.2	4.0	7.5	22.4	64.2
Have not used mifepristone before for any indication	14.4	0.0	15.3	27.2	11.2	10.8
Unfamiliar with clinical data on mifepristone use for EPL	12.7	14.8	16.8	35.0	0.0	0.0
Concerns about being a certified mifepristone prescriber (REMS requirement)	11.0	14.8	0.0	0.0	29.2	17.3
Cannot send mifepristone prescription to pharmacy for dispensing (REMS requirement)	8.7	6.7	14.6	12.1	8.1	0.0
No “champion” to navigate the process	8.1	0.0	23.4	5.4	0.0	0.0
Difficult to stock mifepristone at clinic	7.1	6.6	5.6	7.5	18.0	0.0
Concerns about mifepristone’s association with abortion	6.2	0.0	20.2	0.0	0.0	0.0
No access to recommended protocols for mifepristone use for EPL	3.5	9.2	0.0	5.3	0.0	7.8
Patients have not requested mifepristone	3.3	14.8	0.0	0.0	11.2	0.0
Other reason	1.8	24.0	0.0	0.0	0.0	0.0
Uncertainty with managing complications associated with the medical management of EPL	0.0	0.0	0.0	0.0	0.0	0.0
Concerns about having the patient sign a required consent form from the mifepristone manufacturer (REMS requirement)	0.0	0.0	0.0	0.0	0.0	0.0

Data weighted to account for differential nonresponse by provider sex, region, and years in independent practice. Western Massachusetts is not in this table because all responding obstetrician–gynecologists in the Western region (*n* = 7) have used mifepristone for miscarriage management.

EPL, early pregnancy loss; FDA, Food and Drug Administration; REMS, risk evaluation and mitigation strategy.

### Odds of mifepristone use for miscarriage management and abortion care

Multivariate analysis examined the odds of mifepristone use for miscarriage management and abortion care according to region and controlling for provider sex and practice type (Table 5). Controlling for provider sex and practice type, the odds of using mifepristone for miscarriage management were 86% lower among obstetrician–gynecologists outside of Boston (*i.e.,* in Western, Northeast, Central, and Southeast regions) compared to obstetrician–gynecologists in Boston (aOR = 0.14, 95% CI = 0.08, 0.25; *p* < 0.001). The odds of using mifepristone for abortion care were 54% lower among obstetrician–gynecologists outside of Boston (*i.e.,* in Western, Northeast, Central, and Southeast regions) compared to obstetrician–gynecologists in Boston (aOR = 0.46, 95% CI = 0.26, 0.82; *p* = 0.008).

**Table 5.** Odds of Mifepristone Use for Miscarriage and Abortion Among Obstetrician–Gynecologists in Independent Practice in Massachusetts in 2021, According to Region, Provider Sex, and Practice Type (*n* = 142)

Variable	Odds ratio [95% confidence interval]	
	Mifepristone use for miscarriage management	Mifepristone use for abortion care
Region		
Boston (ref)	1.00 [--]	1.00 [--]
MetroWest	0.60 [0.32, 1.16]	0.79 [0.44, 1.41]
Other region^a^	**0.14 [0.08, 0.25]**	**0.46 [0.26, 0.82]**
Provider sex^b^		
Male (ref)	1.00 [--]	1.00 [--]
Female	**2.83 [1.62, 4.95]**	**3.55 [2.11, 5.98]**
Practice type		
Academic (ref)	1.00 [--]	1.00 [--]
Private practice^c^	**0.34 [0.16, 0.71]**	**0.40 [0.21, 0.74]**
Other^d^	**0.11 [0.05, 0.22]**	**0.18 [0.10, 0.33]**

Bolded values are statistically significant at alpha <= 0.05.

^a^ Other region comprises Western, Northeast, Central, and Southeast regions.

^b^ Provider sex based on the respondent’s answer to their sex assigned at birth as on their original birth certificate.

^c^ Private practice comprises private practice—solo and private practice—group.

^d^ Other practice type comprises health maintenance organization/staff model/non-university hospital, federally qualified health center, community health center or freestanding clinic, and other.

Data weighted to account for differential nonresponse by provider sex, region, and years in independent practice.

## Discussion

Geographic disparities in obstetrician–gynecologists’ use of mifepristone for miscarriage management and abortion care exist in Massachusetts. Providers in Boston, MetroWest, and Western regions reported higher use of mifepristone for both indications while respondents in Southeast, Central, and Northeast regions reported lower mifepristone use. Mifepristone use was also higher for miscarriage management than for abortion care in nearly all regions, despite the lack of FDA labeling for early pregnancy loss. While we did not explicitly ask about barriers to mifepristone provision for abortion care, this finding may suggest that logistical challenges, regulatory requirements, or institutional policies limit mifepristone use for abortion care. This interpretation aligns with prior research indicating that while individual providers may be personally supportive of administering mifepristone for abortion care, organizational or administrative constraints hinder its use.^30^ For example, leaders may perceive that providing mifepristone for abortion care presents challenges with obtaining clinic funding or results in legal action due to changing legislation around medication abortion.

Restrictions like the Hyde Amendment, which bans the use of Medicaid funds for nearly all abortions, may also hinder mifepristone provision for early pregnancy loss. Although only 6.2% of respondents reported that mifepristone’s association with abortion was a barrier to using it for miscarriage management, about one in five respondents (20.2%) in the MetroWest region reported this as a barrier, thus suggesting within-state differences among obstetrician–gynecologists practicing in Massachusetts. This barrier in Massachusetts, where abortion is legal and 16- and 17-year-olds do not need parental consent to obtain abortion care,^31^ is likely amplified in states with stricter abortion laws.

In addition to respondents in Western, Boston, and MetroWest regions reporting higher use of mifepristone for miscarriage management and abortion care, these respondents also tended to report fewer barriers to using mifepristone for miscarriage management. For example, nearly two-thirds of respondents (64.2%) in the Southeast region were unsure how to comply with the FDA REMS regulations compared to just 4.0% of respondents in the MetroWest region. Yet respondents in high-use regions also had unique barriers to mifepristone provision for miscarriage management, such as having a greater proportion of patients not requesting mifepristone (14.8% in Boston) or not having a clinical champion to navigate the process (23.4% in MetroWest). Barriers notwithstanding, these findings overall suggest that patients living in metropolitan Boston, MetroWest, and Western Massachusetts have greater access to mifepristone for miscarriage management and abortion care—with patients living in other parts of the state having more limited access.

The impact of limited access to mifepristone for miscarriage management and abortion care may worsen health inequities by disproportionately impacting individuals already experiencing structural barriers to reproductive health care, including those living in rural areas, young people, and people with racialized identities. In some cases, access is lacking in geographic regions where these individuals disproportionately reside.^32^ For example, two of the five public universities in Massachusetts that are located in “abortion deserts”—areas where in-clinic abortion services are more than 50 miles away—are in the Southeast region,^33^ where we found no obstetrician–gynecologists used mifepristone for abortion care. Young people experiencing intersecting forms of structural oppression including ageism, classism, and other drivers of social inequality may face even greater affordability and accessibility concerns. People with racialized identities are also disproportionately impacted by limited mifepristone access in the state. For example, the Northeast region has a higher proportion of Hispanic adults and fewer English-only speaking households compared to other regions.^34^ With fewer than a third of obstetrician–gynecologists in the Northeast region using mifepristone for miscarriage management and abortion care, it is likely that these geographic disparities in access—combined with structural barriers like racism—will worsen inequities in reproductive health outcomes among racialized groups in the state.

Thus, while Massachusetts is considered a “high access” state with 87% of women living in counties with at least one abortion provider,^24^ targeted interventions are needed to address geographic disparities in mifepristone use for miscarriage management and abortion care. Providing continuing education to clinicians practicing in areas with low mifepristone use (*i.e.,* Southeast, Central, and Northeast Massachusetts) could increase provider comfort with mifepristone. Offering opportunities to review and share protocols and discuss managing potential complications could increase access across the state. Learning collaboratives that educate providers and staff about the clinical uses of mifepristone for early pregnancy loss may also enhance access in regions with low mifepristone use.^30^ Leveraging mentorship can help in enacting clinical and organizational changes.^35,36^ Establishing a mentorship or coaching program that matches clinicians from high-use regions like Boston with those in lower-use regions like Southeast Massachusetts may help clinicians navigate the regulatory complexities and overcome barriers to mifepristone provision.

Interventions for specific practice settings might also help increase access to mifepristone. Consistent with other studies reporting that academic medical centers lead health system innovation,^37^ we found that academic medical centers had higher odds of offering mifepristone for miscarriage management and abortion care, after controlling for provider sex and region, and were clustered in urban areas like Boston. In contrast, we found that other practice types like community health centers and FQHCs had lower odds of mifepristone use and were more geographically dispersed across the state. While community health centers and FQHCs are often underfunded, underresourced, understaffed, and at capacity, they play a crucial role as a safety net for individuals facing financial barriers to care.^38^ As such, targeted investments to integrate mifepristone into these practice types may increase access to mifepristone for patients accessing care outside of academic medical centers, thus facilitating more equitable access to reproductive health care.

Finally, consistent with prior research reporting that obstetrician–gynecologists describe the “REMS program as ‘frightening,’ ‘red tape,’ or ‘[unsure it was] worth the added hassle’”^17^ our study showed that the primary barrier to mifepristone use for early pregnancy loss was uncertainty with how to comply with the FDA REMS regulations, with respondents across all regions reporting this as a barrier to care. Specific aspects of the REMS regulations were also cited as barriers, including concerns about being a certified mifepristone prescriber and inability to send a mifepristone prescription to the pharmacy for dispensing. Because the REMS requirements are considered medically unnecessary by experts in the field,^39,40^ removal of these requirements may increase access to mifepristone for miscarriage and abortion care in Massachusetts and nationwide.

A couple of limitations of this study should be noted. While our response rate aligns with response rates of population-based surveys of physicians,^41^ it is possible that nonresponse bias influenced our estimates. To account for this potential bias, we applied survey weights to account for differential nonresponse by provider sex, region, and experience level. However, it is possible that there were unmeasured factors that differed between responders and nonresponders. We also did not account for obstetrician–gynecologists from the same practice setting taking the survey. Since patients present to an individual clinician, we thought it was worthwhile to measure the number of clinicians who use mifepristone for care. Additionally, we focused solely on barriers to mifepristone for early pregnancy loss, assuming these barriers would also apply to the more stigmatized abortion care. However, it is possible that there are additional, unique barriers specific to mifepristone for abortion care that were not addressed in our survey. Finally, given abortion is codified in Massachusetts law, the barriers facing obstetrician–gynecologists in states more hostile to abortion may differ.

## Conclusion

Obstetrician–gynecologists’ use of mifepristone for miscarriage management and abortion care varies significantly by Massachusetts region. Because patients living in regions where providers do not use mifepristone may only have access to less efficacious medication management and therefore face higher risk for subsequent procedures, variations in mifepristone provision in Massachusetts can lead to regional disparities in reproductive health outcomes. These disparities may worsen existing health inequities by disproportionately impacting individuals already experiencing structural barriers to reproductive health care.

## Abbreviations Used

aOR: adjusted odds ratio
CI: confidence interval
EOHHS: Executive Office of Health and Human Services
EPL: Early pregnancy loss
FDA: Food and Drug Administration
FQHC: Federally Qualified Health Center
HMO: Health Maintenance Organization
REMS: Risk Evaluation and Mitigation Strategy
